# Licosin, a multifunctional defensin peptide originated from the clinical fungus *Lichtheimia corymbifera* with antibacterial and potassium ion channel blocking effects

**DOI:** 10.3389/fmicb.2026.1808106

**Published:** 2026-05-11

**Authors:** Weijia Chen, Yan Yu, Yue Wu, Xinru Chen, Bingzheng Shen

**Affiliations:** 1 Department of Pharmacy, Renmin Hospital, Wuhan University, Wuhan, China; 2 Department of Gastroenterology, Tongji Hospital of Tongji Medical College, Huazhong University of Science and Technology, Wuhan, China; 3 Department of Clinical Pharmacy, School of Pharmaceutical Sciences, Wuhan University, Wuhan, China

**Keywords:** antibacterial activities, CS-α/β structural motif, fungal defensin, *h*Kv1.3 channel inhibitor, licosin

## Abstract

**Introduction:**

Defensins are small, cationic, cysteine-rich peptides that serve as key components of the innate immune system. Here, a new toxin-like fungal defensin named licosin was characterized from the clinical pathogen *Lichtheimia corymbifera*.

**Methods:**

The analysis of its genomic and mRNA sequences revealed that the cDNA sequence of licosin was 207 bp without introns. The deduced precursor peptide of licosin contained 68 amino acids, composed of three parts: an N-terminal signal domain of 20 residues, a pro-peptide of 7 residues that ended at arginineserine, and a mature peptide of 41 residues at the C terminus. The amino acid sequence of mature licosin was further identified by LC-ESI-Q-TOF-MS/MS at the protein level. The mature licosin can form three intramolecular disulfide bonds. Circular dichroism spectrum analysis and 3D homology modeling revealed that mature licosin can form the representative cysteine-stabilized *α*-helical and *β*-sheet (CS/αβ) superfamily.

**Results:**

Like most defensins, licosin exhibited good antimicrobial activities against gram-positive bacteria, including clinical isolates of MRSA and *Micrococcus luteus*. Interestingly, similar to animal toxins, licosin also exhibited potassium ion channel blocking activities. It displayed a dose dependent selective inhibition of the immune-related human potassium channel (*h*Kv1.3) with an IC50 value of 0.4 ± 0.06 μM. Two lysine residues at positions 34 and 35, with strong positive charges, played a decisive role in the licosin-*h*Kv1.3 channel interaction.

**Discussion:**

These findings suggest that the active peptide secreted by clinical pathogenic fungi could be a treasure trove for the discovery of peptide-based drugs.

## 1 Introduction

To date, more than 21,000 species of fungi have been characterized (Hibbett et al., 2025), of which approximately one hundred strains are pathogenic fungi (Richardson and Warnock, 2012). In recent years, fungal infections have led to over 1.6 million deaths annually, and more than one billion people have suffered from severe fungal diseases (Almeida et al., 2019). Fungi can cause a variety of sicknesses, such as asthma, allergies, rashes, skin and nail infections, fungal pneumonia, and fungemia (Denning, 2024). Moreover, the incidence of opportunistic fungal infections in patients with solid or hematologic malignancies or those receiving organ transplants has dramatically increased in recent years (Ruiz-Camps and Aguilar-Company, 2021; Lionakis et al., 2023; Popova and Rogacheva, 2025).

During pathogen–host interaction, bioactive agents with immunosuppressive effects produced by pathogenic fungi to resist the host’s innate immunity are necessary for their successful infection and persistence in humans. The fungal defensins, a group of small cysteine-rich cationic antimicrobial peptides containing three to four pairs of intramolecular disulfide bonds (Gani et al., 2025; Oliveira Júnior et al., 2025; Roque-Borda et al., 2025), were discovered in recent decades from *Pseudoplectania nigrella* (Plectasin), *Eurotium amstelodami* (Eurocin), *Microsporum canis* (Micasin), *Coprinopsis cinerea* (Copsin), *Trichophyton interdigitale* (Triintsin), and *Purpureocillium lilacinum* (Purlisin-CT) (Mygind et al., 2005; Oeemig et al., 2012; Zhu et al., 2012; Essig et al., 2014; Shen et al., 2018, 2020; Chen et al., 2025). All these peptides with antimicrobial properties adopt a cysteine-stabilized *α*-helix and *β*-sheet (CSαβ) structural motif, which has a conserved cysteine scaffold also widely found in animal toxins (Zhu et al., 2011; Yerramsetty et al., 2022).

Here, we report a multifunctional fungal defensin from a clinical fungus. The strain was isolated from a male patient with early stomach cancer after receiving endoscopic mucosal resection (EMR) and endoscopic submucosal dissection (ESD). The isolate of *Lichtheimia corymbifera* was identified by transcriptome sequencing and sequence alignment. Through combined sequence analysis and molecular cloning, the gene and cDNA sequences of a novel fungal defensin, termed licosin, were discovered. The full-length licosin deduced from its cDNA sequence is composed of 68 amino acid residues, and its existence was further verified in the extraction of total protein by liquid chromatography–electrospray ionization–quadrupole–time-of-flight tandem mass spectrometry (LC-ESI-Q-TOF-MS/MS). The results of bioinformatic analysis indicated that the precursor defensin of licosin contains three parts: an N-terminal signal domain of 20 residues, a precursor peptide of 7 residues that ends with lysine–arginine, and a mature peptide of 41 residues at the C terminus. The mature licosin shows a typical CSαβ scaffold via three intramolecular disulfide linkages. Pharmacological studies revealed that licosin possesses multiple biological functions, including antimicrobial activities and selective potassium ion channel inhibitory effects. Our findings suggest that the fungal defensin licosin might be a promising bio-drug candidate for antibacterial agents or immunomodulators. More importantly, it highlights that bioactive peptides secreted by human pathogenic fungi are a valuable resource for new drug discovery.

## 2 Materials and methods

### 2.1 Isolation and identification of human pathogenic fungus

Human clinical samples were collected from a 54-year-old woman with gastric cancer who had received EMR and ESD treatments. The lesion was located at the patient’s right root. After debridement of infected sites and decontamination of surrounding normal skin, sterile disposable swabs were used to extract secretions and collect partial erosive tissue, which were then immediately placed into a sterile centrifuge tube containing 2.0 mL of 0.9% sterilized normal saline (NS). Following vortex mixing and centrifugation at 10,000 rpm for 15 min at 4 °C, the upper solution was removed. Approximately 800 μL of the supernatant was evenly spread on a 15 cm diameter potato-dextrose (PDA) solid medium dish containing 0.25 mg/mL chloramphenicol. The cultural characteristics of the clinical isolates were observed on the PDA medium plate at 28 ± 0.5 °C for 10 days. To obtain the pathogenic fungi in pure culture, a single colony of the dominant strain was selected and inoculated into a new 9 cm diameter PDA culture plate. The clinical isolate was initially characterized using microscopic examination and biochemical methods by the clinical laboratory of Renmin Hospital affiliated with Wuhan University. The fungal genomic DNA was extracted using the Fungi/Yeast Genomic DNA Isolation Kit (Sangon Biotech, Shanghai) following the manufacturer’s instructions. Total RNA was obtained using TRIzol reagent (Takara Biotechnology, Dalian, China). The quality of total RNA was visually inspected through 1% agarose gel electrophoresis. The purity, represented by the ratio of absorbance at 260 and 280 nm, was quantified using a Nanodrop ND-1000 spectrophotometer (Thermo Scientific, Wilmington, DE). To remove residual DNA, total RNA was treated with the RQ1 RNase-Free DNase Kit at 37 °C for 20 min (1 unit/μL) (Promega, Madison, WI, USA) following the manufacturer’s protocol, and then stored in RNase-free water at −80 °C. The cDNA libraries of the clinical fungus *L. corymbifera* were established, and high-throughput sequencing was carried out by Biomarker Cloud Technologies Co., Ltd. (Wuhan, China). After separation and purification of agarose gel, the fractions between 150 bp and 250 bp were selected, and these cDNA fragments were ligated with the sequencing adapters. Subsequently, the library was added to a flow cell and bridge PCR was conducted to amplify DNA fragments as single DNA molecule clusters. The HiSeq TM 2000 high-throughput platform was used for transcriptome sequencing, and Illumina software was used to analyze the data. The transcriptome data of this pathogenic isolate were mapped to the NCBI genome database of the corresponding strain.

### 2.2 Cloning and bioinformatics analysis

The clinical fungi were cultured at 28 °C for 10 days on a 9 cm diameter sterile PDA plate supplemented with 0.25 mg/mL of chloramphenicol. The colonies, including spores and aerial mycelia, were scraped off the agar plate using a sterile loop and ground with liquid nitrogen. The purified total RNAs were reverse-transcribed with a poly(T) adapter into first-strand cDNAs using the PrimeScript II 1st strand cDNA synthesis kit (Takara, Dalian, China). The first-strand cDNAs served as templates for random primer amplification in PCR reactions. Specific primers were designed according to the protein-coding DNA sequence (CDS) (GenBank accession number: CBTN010000034.1:331743–331,949). To characterize the sequence and structure of the defensin genes in this clinical fungus, fungal genome and cDNAs were used as templates for selective amplification with the following primers: F/R (FP: 5′-ATGGTCAACAAGATTATCG-3′; RP: 5′-TTAGTTGCAGACGCAGACCTTC-3′). Amplification reactions (50 μL) were performed using 30 ng of template DNA, 5 μM of each primer, and Phanta® Super-Fidelity DNA polymerase (Vazyme Biotech, Nanjing, China). Thermal cycling was initiated with a 3 min incubation at 95 °C, followed by 40 cycles of 95 °C for 40 s, 60 °C for 15 s, and 72 °C for 30 s, with a final extension step at 72 °C for 1.5 min. The PCR products were fractionated on a 1.0% (w/v) agarose gel, and selected bands were extracted using the FastPure® Gel DNA Extraction Mini Kit (Vazyme Biotech, Nanjing, China). The purified nucleic acids were blunt-end ligated into the pClone007 Blunt Simple Vector (TsingKe Biotech, Beijing, China). The 20 μL reaction mixture was used directly to transform chemically competent *Escherichia coli* DH5α (Toyobo, Osaka, Japan). All cells were spread on Luria–Bertani (LB) agar plates containing 75 μg/mL of ampicillin to select transformants. Ten colonies were randomly selected, and the positive clones were validated by colony PCR. The recombinant plasmid containing the foreign gene was extracted from a single colony using the PurePlasmid Mini Kit (CWBIO, Beijing, China), and the inserted DNA fragment was sequenced using M13F (5′-TGTAAAACGACGGCCAGT-3′) and M13R (5′-CAGGAAACAGCTATGACC-3′) primers.

The putative cleavage site of the signal peptide in full-length licosin was predicted using the SignalP 5.0 server^1^ (Almagro Armenteros et al., 2019). Using the NCBI database with the BLAST program (Boratyn et al., 2013), representative sequences derived from fungi were screened and selected to carry out multiple sequence alignments using Clustal Omega (Madeira et al., 2024), which included different defensin peptides derived from fungi, plants, insects, and primates. Based on the results of the sequence alignments, a phylogenetic tree was constructed using MEGA 12 software with 1,000 bootstrap replicates (Kumar et al., 2024).

### 2.3 Verification of fungal defensin licosin at the protein level

The pathogenic fungus *L. corymbifera* was inoculated on a sterile PDA plate. After 7 days of culture, ~ 300 mg of mycelium was scraped from the media. The collected fungi were ground in liquid nitrogen 3 to 5 times, then snailase and dl-dithiothreitol (DTT) were added to degrade the fungal cell walls. PBS buffer (30 mmol/L, pH 7.8) was immediately added to bring the total volume to 20 mL. The sample was vortexed twice for 1 min to mix thoroughly, incubated at 37 °C for 4 h, and then centrifuged at 14000 rpm for 5 min at 4 °C. The supernatant was discarded, and 1 mL of ice-cold protein lysis buffer containing 8 mol/L urea and 200 mmol/L sodium carbonate was added to the centrifuge tube. The precipitate was resuspended and shaken vigorously for 20 min at 4 °C. The total protein extracted from *L. corymbifera* was desalted and lyophilized. The freeze-dried protein powders were dissolved in 100 μL of Milli-Q water. For efficient separation and enrichment, reverse-phase high-performance liquid chromatography (RP-HPLC) was performed on an Agilent 1,260 system (Palo Alto, CA, USA). A total of 100 μL of protein solution was injected into an ODS C18-BP reversed-phase column (Elite, 4.6 × 250 mm, 5 μm) and eluted with solvent A (0.15% (v/v) TFA in aqueous solution) and solvent B (100% (v/v) acetonitrile, 0.15% (v/v) TFA). The flow rate was 1.0 mL/min with detection at 225 nm using a UV detector. The optimized chromatographic conditions were as follows: isocratic (95% solvent A) from 0 to 10 min, a gradient to 95% solvent B from 10 to 70 min, and isocratic (95% solvent A) from 70 to 90 min. The fractions corresponding to each chromatographic peak were collected separately and lyophilized. Ten μL of trypsin solution (Worthington trypsin, 100 ng/mL in 1 mM HCl) was added to 90 μL of protein solution (~ 25 ng) at a trypsin: protein ratio of 1:50 (w/w). After the reaction system was incubated at 37 °C overnight, DTT was added to achieve a final concentration of 30 mM and then incubated at 40 °C for 45 min. The sample was cooled to room temperature and mixed with iodoacetamide at a final concentration of 40 mM, subsequently incubated in the dark for another 30 min. The specimen was then cooled to room temperature and alkylated for 60 min with iodoacetamide at a final concentration of 100 mM in the dark. Prior to mass spectrometry (MS), nanoscale packed-capillary liquid chromatography (LC) was conducted using a Dionex Ultimate 3,000 LC system equipped with a trap column (Acclaim PepMap100 C18, 75 μm × 15 cm). The column was coupled to a quadrupole–time-of-flight mass spectrometer (Q-TOF MS) (microTOF-Q II, Bruker Daltonics, USA) in positive ion MS mode. Collision-induced dissociation (CID) was used to generate fragment ions for further detection, with the collision energy set to automatic mode and an ion spray voltage of 2.8 kV at a heated capillary temperature of 220 °C. The full scan mass range was 100 to 5,000 Da at an Orbitrap resolution of 30,000. A blank sample was run for instrument control, based on m/z ratio and charge number. The MASCOT engine (Matrix Science, London, United Kingdom; V 2.3.0) was used to extract the raw data file into a set of DTA files and to search the MS/MS data of peptides against fungal protein and transcriptomic databases from NCBI. A mass tolerance of 5 ppm for precursor ion scans and a mass tolerance of 0.5 Da for product ion scans were used. Scaffold version 4.4.3 (Proteome Software Inc., Portland, OR) was utilized to calculate spectral counts and validate peptide/protein identifications, with a confidence level of 95%.

### 2.4 Preparation and molecular weight determination of mature licosin

The linear mature defensin (reduced form, licosin-Re) was obtained via solid phase synthesis by GL Biochem (Shanghai, China) Ltd. or DGpeptides Co., Ltd. (Wuhan, Hubei, China) with a purity of more than 95%. The intramolecular disulfide bonds were paired through oxidation in refolding buffer. One milligram of linear peptide was dissolved in 2 mL of 0.15 M Tris–HCl buffer (pH 7.8). The pairing reaction occurred spontaneously at room temperature (RT) for 48 h and was promoted by continuous gentle shaking at 80 rpm. The reaction solution was centrifuged at 10000 g for 5 min at 4 °C, followed by desalination and purification using RP-HPLC.

The molecular weight of licosin was initially confirmed by SDS-PAGE. The precise molecular mass of the fungal defensin with intermolecular disulfide bonds (oxidized form, licosin-Ox) was determined by matrix-assisted laser desorption ionization time-of-flight (MALDI-TOF) mass spectrometry. The salt-free licosin-Ox solution was mixed with 200 μL of MALDI-matrix (15 mg/mL *α*-cyano-4-hydroxycinnamic acid (CHCA), 0.1% TFA, and 40% acetonitrile). Subsequently, 1 μL of the peptide sample mixture was spotted onto a MALDI stainless steel sample plate and allowed to air-dry. To obtain the molecular weight of the fungal defensin licosin-Ox, mass spectrometry analysis was carried out, selecting a mass range between 1,000 and 10,000 m/z (charge-to-mass ratios) to collect the signals using the AutoXecute tool of FlexControl acquisition software (Version 3.0, Bruker-Daltonics), with the accelerating voltage set to 25 kV in positive-ion reflection mode. The reproducibility of the spectrum was checked multiple times from separately spotted samples.

### 2.5 Structure exploration of mature licosin

After the formation of intermolecular disulfide linkages, the secondary structures of fungal defensin licosin were studied by circular dichroism (CD) spectroscopy, recorded on a Jasco J-810 spectropolarimeter (JASCO International Co., Ltd., Japan). Licosin was dissolved in Milli-Q water at a concentration of 200 μg/mL. Using a cell with a 1 cm path length, CD spectra were collected at room temperature from 185 to 260 nm with a wavelength resolution of 0.1 nm and an accumulation of 3 scans. The scanning speed was 50 nm/min, and the response time was set to 1 s. The results were presented as mean residue weight molar ellipticity [*θ*] (deg·cm^2^·dmol^−1^). [θ] was defined as θ_millidegrees_/(10 × number of amino acid (*lc*)). *l* the length of the light path, and *c* was the concentration of the peptide to be tested. All measurements were repeated three times, and the data were recorded and processed by the JASCO software.

The spatial structure of fungal defensin licosin was predicted and analyzed using the SWISS-MODEL workspace^2^, with insect defensin as the template (PDB:2LLD) (Bienert et al., 2017; Waterhouse et al., 2018).

### 2.6 Assessment of antibacterial activity

The antimicrobial function, a common property of defensins, has been evaluated. The reference strains as well as clinical isolates were utilized to explore the antibacterial activities of licosin. *Staphylococcus aureus* ATCC6538, Methicillin-resistant *Staphylococcus aureus* (MRSA) ATCC 43300, *Staphylococcus epidermidis* AB208187, *Micrococcus luteus* ATCC 4698, *Escherichia coli* AB94012, *Acinetobacter baumannii* ATCC 19606, and *Klebsiella pneumoniae* ATCC 31314, were purchased from the China Center of Type Culture Collection (CCTCC). Pure cultures of two identified clinical isolates, methicillin-resistant *S. aureus* (MRSA) and *Micrococcus luteus*, were obtained from the Clinical Laboratory of Renmin Hospital attached to Wuhan University. The antimicrobial effects quantified by the use of minimum inhibitory concentrations (MIC) values were determined according to the Clinical and Laboratory Standards Institute two-fold serial microdilution methods (Clinical and Laboratory Standards Institute, 2018). The strains stored on agar slants at 4 °C were inoculated onto solid LB medium dishes and incubated at 37 °C overnight for activation. A single colony was selected and used to expand cultivation in 10 mL of liquid LB medium. An overnight-incubated bacterial inoculum (turbidity equivalent to the McFarland standard of 0.5) was prepared in a test 96-well plate (about 5 ~ 10 × 10^7^ CFU/mL). Twenty microliters of serial two-fold dilutions of the defensin were made and mixed with 130 μL of bacterial culture, resulting in a total volume of 150 μL, with final concentrations of licosin peptide ranging from 128 μM to 1 μM. Ampicillin was used as a positive control. The 96-well plates were secured with rubber bands and then agitated on a rotary shaker to mix the peptide solution and bacterial liquid suspension well. The mixture was incubated at physiological temperature (36 ± 1 °C) for 24 h, and the optical densities at 630 nm (OD630) were measured by a microtiter optical plate reader (Multiskan FC, Thermo Fisher Scientific, USA) to determine the MIC values. All these experiments were performed in three parallel tests.

### 2.7 Scanning electron microscope and time-kill curve

Overnight-cultured MRSA ATCC 43300 was transferred to LB medium and cultured to the exponential phase. Three hundred microliters of the licosin peptide solution was added to a 1,200 μL bacterial suspension. The final concentration of licosin was 16 μM, and the mixture was incubated at 37 °C with continuous shaking. After 30 min of incubation, the bacterial suspension was centrifuged at 1000 × g for 5 min, and the pellet was washed with 0.1 M phosphate-buffered saline (PBS) several times and then fixed overnight with 2.5% glutaraldehyde in 0.1 M PBS at 4 °C. After fixation, the bacteria were washed with PBS for a minimum of 15 min and then dehydrated using a series of graded ethyl alcohols (50% for 15 min, 60% for 15 min, 70% for 15 min, 80% for 15 min, 90% for 15 min, and 2 changes of 100% for 10 min each). After this, the samples were mounted on aluminum stubs with adhesive tabs and sputter-coated for 3 min using a Polaron. The samples were observed on the Hitachi X650 scanning electron microscope (SEM).

### 2.8 Time-kill curve

The active bacteria (*S. aureus* ATCC6538) were diluted to approximately 3.5 log CFU/mL with 0.01 M phosphate buffer solution (PBS) and then mixed with licosin or ampicillin at a final concentration of 1 × MIC. Twenty microliters of samples were plated evenly on solid LB medium plates and then cultured at 37 °C for 24 h at different time intervals (0, 1, 2, 3, 4, and 5 h). The diluted bacteria mixed with ampicillin were treated as the positive control group.

### 2.9 Electrophysiological recordings of potassium channels and binding model between licosin and *h*Kv1.3 channel

Human embryonic kidney 293 T (HEK293T) cells, obtained from the American Type Culture Collection, were grown at (36 ± 0.5) °C in 5% CO2/95% humidified air in Dulbecco’s modified Eagle’s medium (DMEM) supplemented with 15% fetal bovine serum (FBS) (Gibico, CA, USA), 75 U/mL penicillin, and 75 μg/mL streptomycin. The nucleotide sequences of different potassium channel subtypes, including *h*Kv1.1, *h*Kv1.2, and *h*Kv1.3, were provided by Prof. Yingliang Wu, College of Life Science, Wuhan University, China. The coding sequences were then amplified by PCR and subcloned into the eukaryotic expression vector pIRES2-EGFP (Clontech, Palo Alto, CA). These recombinant plasmids were transfected into HEK293 cells to co-express the channel protein and enhanced green fluorescent protein (EGFP) reporter using Lipofectamine 2000 transfection reagent (Invitrogen, USA), in accordance with the manufacturer’s protocols. The positive cells were selected by observing green fluorescence under a fluorescence microscope (ICX41, Shunyu, China) after 12 to 36 h of transfection for electrophysiological tests.

Potassium channel current recordings were carried out at room temperature using an EPC 10 patch clamp amplifier (HEKA Elektronik, Lambrecht, Germany). The recording chamber was continuously perfused with a multichannel micro-perfusion system (MPS-2, INBIO Inc., China). Patch pipettes were fashioned from borosilicate glass capillary tubing using a two-stage puller (Narishige PC-100 micropipette puller, Tritech Research, Japan) and fire-polished to a final resistance of 2–5 MΩ. The pipette was positioned close to a single cell via a micromanipulator (MP-225, Sutter Instrument). A small negative pressure was applied to the pipette to facilitate tight seal formation (1–5 GΩ). To monitor the dynamic changes in cell membrane current, whole-cell voltage-clamp recordings were performed online using PatchMaster (Heka Elektronik, Germany). The external mammalian Ringer’s solution contained (in mM): 5 KCl, 140 NaCl, 5 HEPES, 2 CaCl2, 1 MgCl2, 10 D-glucose, and was adjusted to pH 7.4 with NaOH. The internal patch pipette solution contained (in mM): 140 KCl, 1 MgCl2, 1 EGTA, 1 Na2ATP, 10 HEPES, and was adjusted to pH 7.4 with KOH. The procedures for current monitoring and data acquisition/analysis for whole-cell recording have been reported previously (Shen et al., 2020). For *h*Kv1.1, *h*Kv1.2, and *h*Kv1.3 channels, whole-cell currents were generated by depolarizing voltage steps of 200 ms from a holding potential of −80 mV to +50 mV at 20 s intervals. All samples to be tested were dissolved in an external solution containing 0.05% BSA to prevent non-specific binding. The leakage current and residual capacitative current were digitally subtracted, and data were processed and analyzed using Origin software (Version 2019b, OriginLab Corp., USA). To maintain the 293 T cells in good condition and ensure stable channel current during the electrophysiological assay, the infusion time of peptides was limited to less than 20 min. The relationship between the dosage of defensin and the suppression effect on potassium current was measured based on the modified Hill equation: I_defensin_/I_control_ = 1/{1 + ([peptide]/IC_50_)}, where I was the peak current and [peptide] was the concentration of fungal defensin licosin. The half maximal inhibitory concentration (IC50) was defined as the peptide concentration that caused 50% of current blockade. All experiments were repeated at least three times (n ≥ 3), and data are presented as mean ± standard errors (S.E).

Molecular simulations were used to explore the binding model and the mechanism ligand–receptor interaction. The homologous 3D structure of the target (*h*Kv1.3 channel) was built as previously described (Jin and Wu, 2007). The complexes of defensin-*h*Kv1.3 channel were generated using sequence analysis tools and the Z-DOCK module, which were integrated into the Discovery Studio software package V3.5 (Accelrys Software Inc., San Diego, CA, USA).

### 2.10 Cellular viability assay

The mouse fibroblast (NIH-3 T3) and human umbilical vein endothelial cells (HUVECs) were used to preliminarily evaluate the safety of licosin peptide at the cellular level for topical and injection applications. The NIH-3 T3 cells were cultured in 10% FBS/DMEM. HUVECs from passages 3 to 8 were cultured in M200/LSGS medium. The cells (~5 × 10^5^) were seeded in a cell culture dish pre-coated with 0.1% gelatin (10 cm, Nest Biotechnology Co.) at 37 °C in a standard humidified incubator with a 5% CO2 atmosphere. After 12 h of culture, the medium was discarded to remove the non-adherent cells, and new complete culture medium was added. Cells were maintained in a 5% CO2 atmosphere at 37 °C until further processing. The medium was replaced every three days. The cellular viability of NIH-3 T3 and HUVECs was measured by the Cell Counting Kit-8 assay according to the manufacturer’s instructions (CCK-8, Beyotime Biotechnology, China). HUVECs (5,000 cells/well) were seeded into 96-well plates and incubated overnight before treatment with various concentrations of licosin (64, 32, 16, 8, 4, 2, or 1 μmol/mL) for 12 h. Subsequently, 10 μL of CCK-8 solution was added to each well, and the cells were incubated for an additional 2 h. The optical density of each well at 450 nm was determined by a microplate reader (Thermo Scientific Multiscan FC).

### 2.11 The influence of acids, bases and salt on the antibacterial activity of licosin

Licosin peptide was dissolved in 0, 50, 100, 150, and 200 mM sterile NaCl solutions, respectively. The MICs of the peptides against *S. aureus* ATCC6538 were determined with increasing salt concentrations. Experiments were performed in triplicate. In addition, the peptide in physiological saline was used as a control. To determine pH stability, licosin was adjusted to a pH range from 3.0 to 10.0 using 0.1 M citric acid–sodium citrate buffer (pH 3.0 ~ 5.0), 0.2 M sodium phosphate buffer (Na_2_HPO_4_-NaH_2_PO_4_) (pH 6.0 ~ 7.0) and 0.05 M Tris–HCl buffer (pH 8.0 ~ 9.0) (Cao et al., 2015). The antimicrobial activity of peptides against *S. aureus* ATCC6538 was determined by MIC assays.

### 2.12 Statistical analysis

All data are expressed as mean ± S. E. Statistical analysis was carried out by using a one-way analysis of variance (ANOVA), along with multiple comparisons tests or Student’s *t*-test, employing GraphPad Prism version 9.0. Significance levels were defined as follows: *p < 0.05, **p < 0.01, and ***p < 0.001.

## 3 Results

### 3.1 Isolation and identification of the clinical fungi *L. corymbifera*

**Figure 1:**
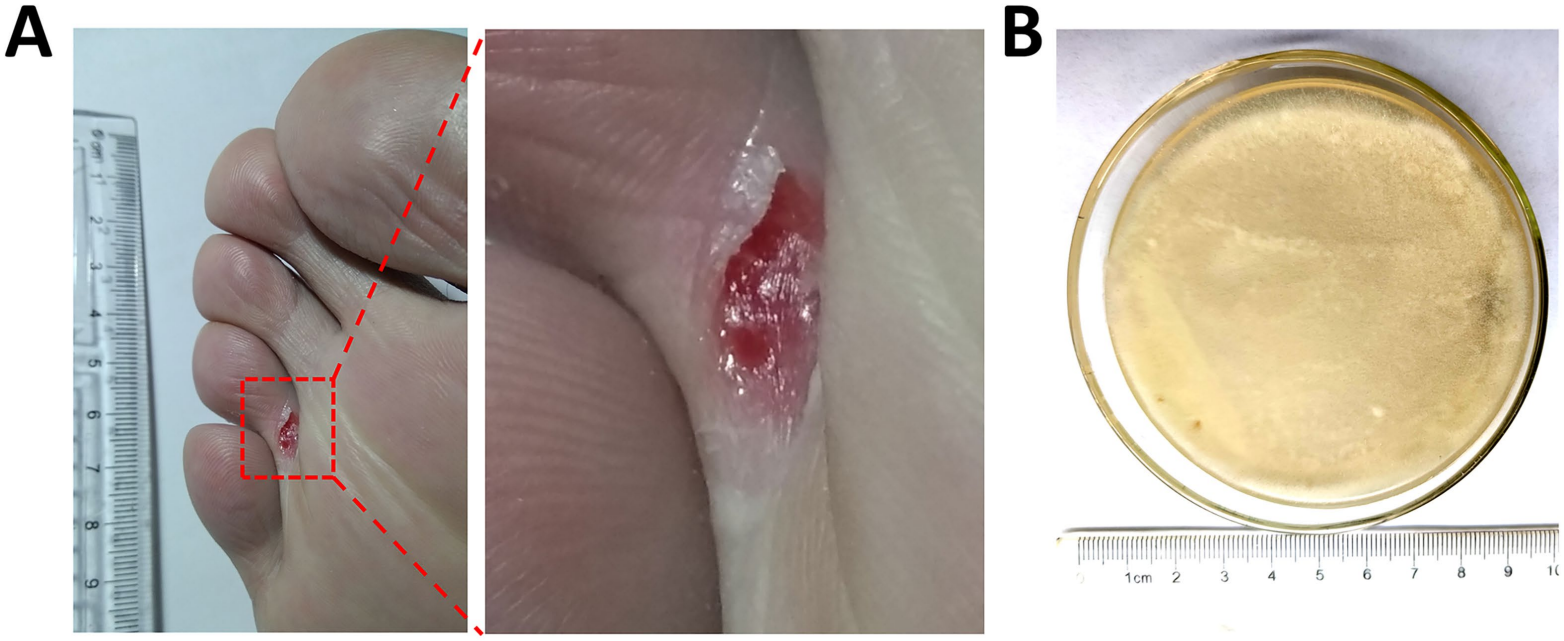
Clinical sample and isolated culture. **(A)** The toe ulcer on the right foot of a 54-year-old woman with gastric cancer. The wound surface area was 1 cm × 0.4 cm with secretion, where the skin had turned a grayish red color. **(B)** Colony morphology of clinical fungi *L. corymbifera* on a solid PDA plate with 0.25 mg/mL chloramphenicol, which displayed wool-like colonies and visible elongated mycelium.

The sample was taken from an inpatient with a toe ulcer (Figure 1A). The secretions located in the central area of the ulcer were drawn out for further analysis. The clinical pathogenic fungi were preliminarily identified as *L. corymbifera* by the clinical laboratory of Renmin Hospital affiliated with Wuhan University (Figure 1B). The total RNA of the fungi *L. corymbifera* was extracted. The rate of OD260/OD280 was measured, and the value was 1.89. The fungal transcriptome sequences were mapped into the whole-genome sets from five different *L. corymbifera* strains in the GenBank database, with coverage rates ranging from 96.23 to 98.38% (Supplementary Table S1). *L. corymbifera* is a member of the Mucoromycotina and was originally described in Poland more than 100 years ago (Schwartze et al., 2014). This fungus is normally found in saprotrophic environments but can also act as an opportunistic pathogen, causing orbital infections (Chandran et al., 2022), subcutaneous mucormycosis (Razouk et al., 2012), or postoperative infections (Eickhardt et al., 2013). *Lichtheimia* species are the third most common cause of mucormycosis worldwide (Schwartze et al., 2014). Most of the infected individuals are patients with immune suppression or other underlying diseases. Consequently, after the EMR/ESD operation, this 54-year-old patient with gastric cancer was likely more susceptible to cutaneous fungal infections caused by *L. corymbifera*.

### 3.2 cDNA of Licosin gene and sequence analysis

**Figure 2:**
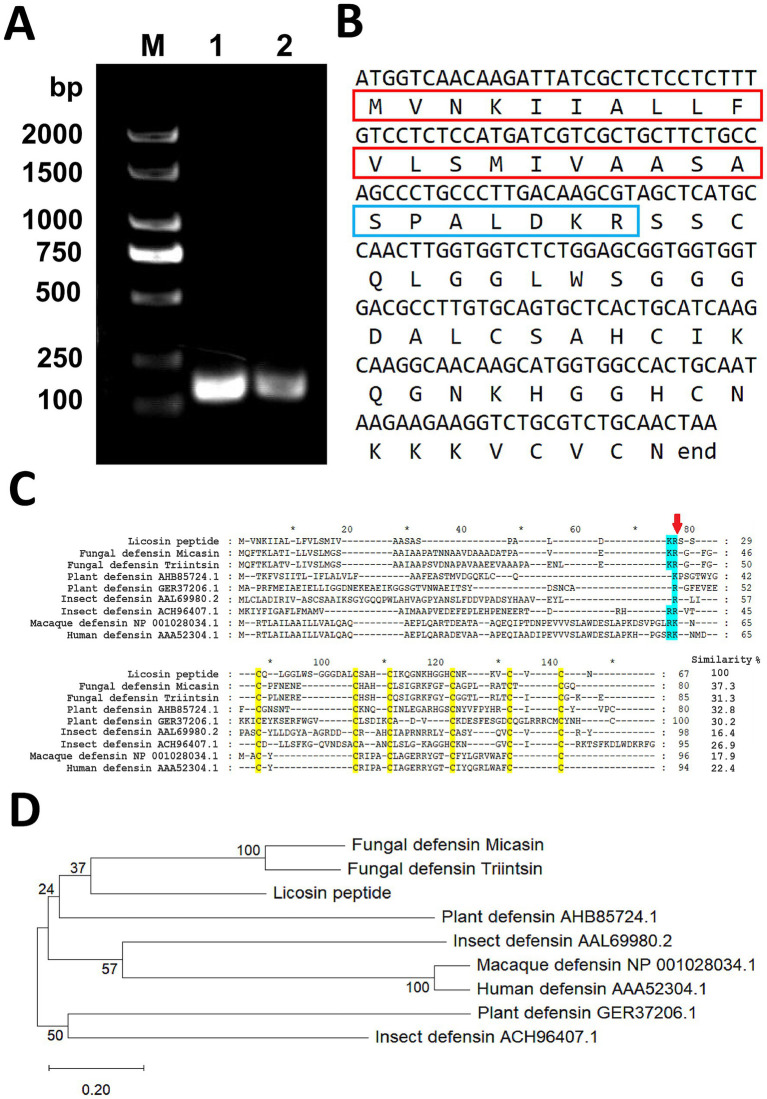
Gene cloning, sequence, and evolution analysis of licosin. **(A)** Amplification of the licosin gene and coding sequence from clinical pathogenic fungi *L. corymbifera*. Lane M: DNA marker; Lane 1: Amplification products using fungal genomic DNA as a template; Lane 2: Amplification products using first-strand cDNA as a template. **(B)** The CDS and the corresponding amino acid sequence of licosin. The putative signal peptide (positions 1–20) and precursor peptide (positions 21–27) are indicated by red and blue rectangle boxes, respectively. **(C)** Sequence alignment of licosin and other peptides from fungi, plants, insects, and primates. The cysteines and basic residues are shaded in brilliant yellow and blue, respectively. The position between the precursor peptide (positions 21–27) and the mature peptide (positions 28–68) is indicated by a red arrow. **(D)** Phylogenetic tree based on the sequence alignment of licosin and other defensin peptides. The percentage of bootstrap support was calculated and displayed on the branch lines.

To characterize the genomic and coding sequence (CDS) of licosin and deduce the corresponding amino acid sequence, genome and first-strand cDNAs reverse-transcribed from total RNAs were used as templates for PCR reactions. Agarose gel electrophoresis of the specific amplification products showed that the bands of licosin genomic DNA and CDS sequences ranged in size from 100 bp to 250 bp (Figure 2A). The results indicated that the gene for licosin contained no introns. The sequence deduced from the CDS contained 68 amino acids, consisting of a putative signal peptide (positions 1–20), a precursor peptide (positions 21–27), and the mature peptide (positions 28–68) at the C-terminal end (Figure 2B). Multiple sequence alignment of representative defensin peptides from fungi, plants, insects, and animals is presented (Figure 2C). Although the values of sequence identity for the full-length protein between different species were remarkably low, all cysteine residues determining their spatial conformation were highly conserved. Among other species, the full-length defensin peptide from the plant *Brassica juncea* (NCBI No. AHB85724.1) exhibited the highest similarity value of 32.8% with licosin. Based on the results of the similarities and multiple sequence alignments, a phylogenetic tree was generated, and the phylogenetic relationships among the various defensins were characterized (Figure 2D).

### 3.3 Separation and identification of licosin peptide from total protein extraction

**Figure 3:**
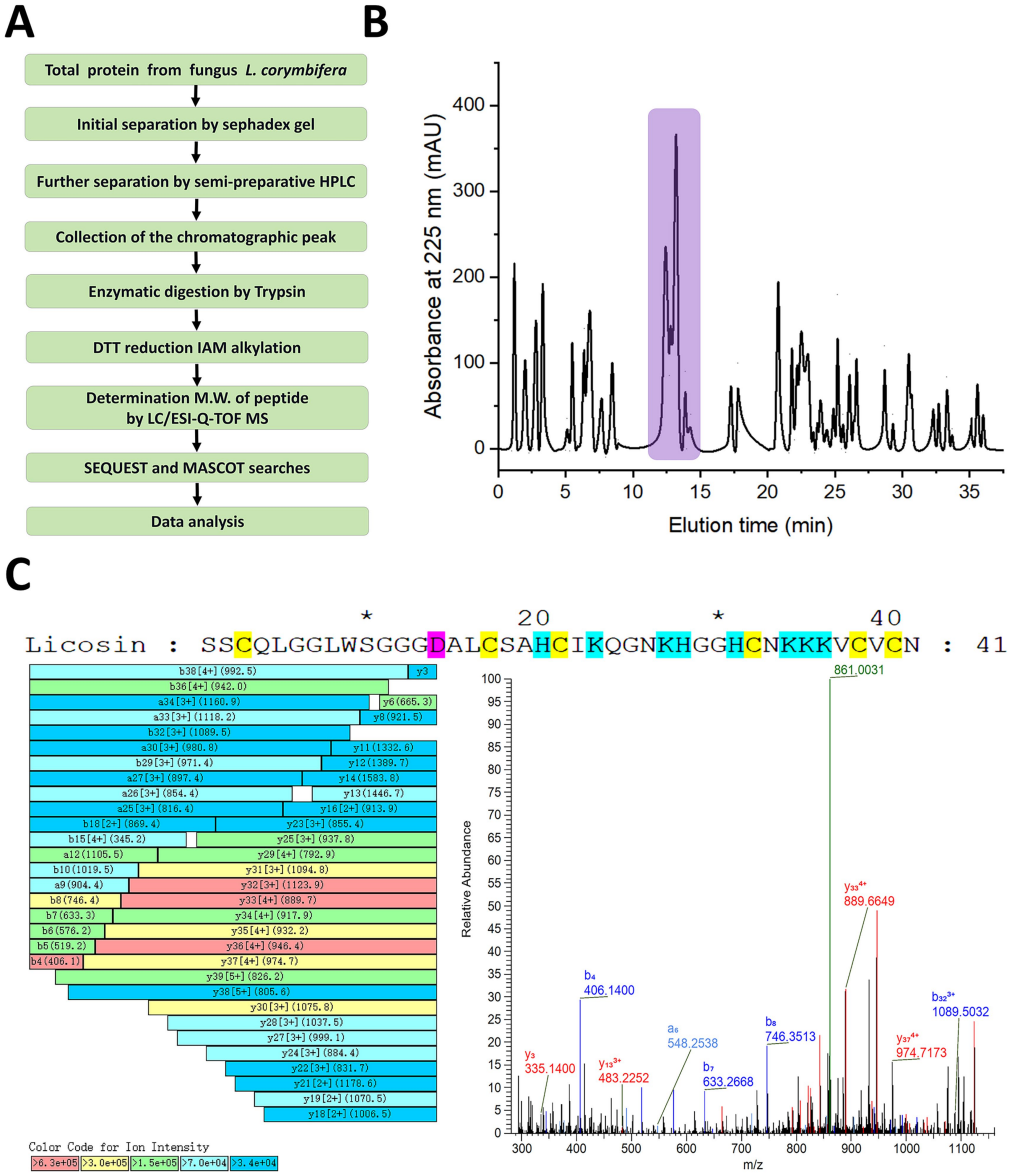
Identification of mature licosin in the total protein extracted from *L. corymbifera*. **(A)** Systematic strategy for proteomic analysis of the total protein from the clinical fungus. **(B)** The separation of protein components with a molecular weight less than 8,000 Da by RT-HPLC. The identified fragment of the mature licosin peptide was from the chromatographic peaks shaded in light purple. **(C)** Identification of licosin by LC-ESI-Q-TOF-MS/MS combined with MASCOT searching. The amino acid sequences were confirmed by analyzing a_n_, b_n_, and y_n_ ions and their derivatives. The cysteines, acidic residues, and basic residues were colored in brilliant yellow, magenta, and blue, respectively.

To verify the presence of the fungal defensin in the pathogenic isolate of *L. corymbifera*, preliminary separation was carried out using Sephadex gel, followed by precise component collection through semi-preparative HPLC. Chromatographic separation techniques combined with LC-ESI-QTOF-MS/MS analysis were employed to validate the existence of the licosin peptide (Figure 3A). Initially, the total protein extraction was filtered through a gel column. The components with molecular weights less than 8,000 Da were further separated by RT-HPLC. Peptides and proteins with different polarities were eluted at varying retention times (Figure 3B). The chromatographic peaks at retention times of ~ 11 to 15 min were collected and digested by trypsin. After reductive alkylation, the fragments of licosin were identified via LC-ESI-Q-TOF-MS/MS and MASCOT engine searching. The identified amino acid sequences were fragments of the mature licosin peptide, which consisted of 41 amino acids and was located at the carboxy terminus of the full-length licosin (position 28–68) (Figure 3C).

### 3.4 Preparation, verification and structure of licosin

**Figure 4:**
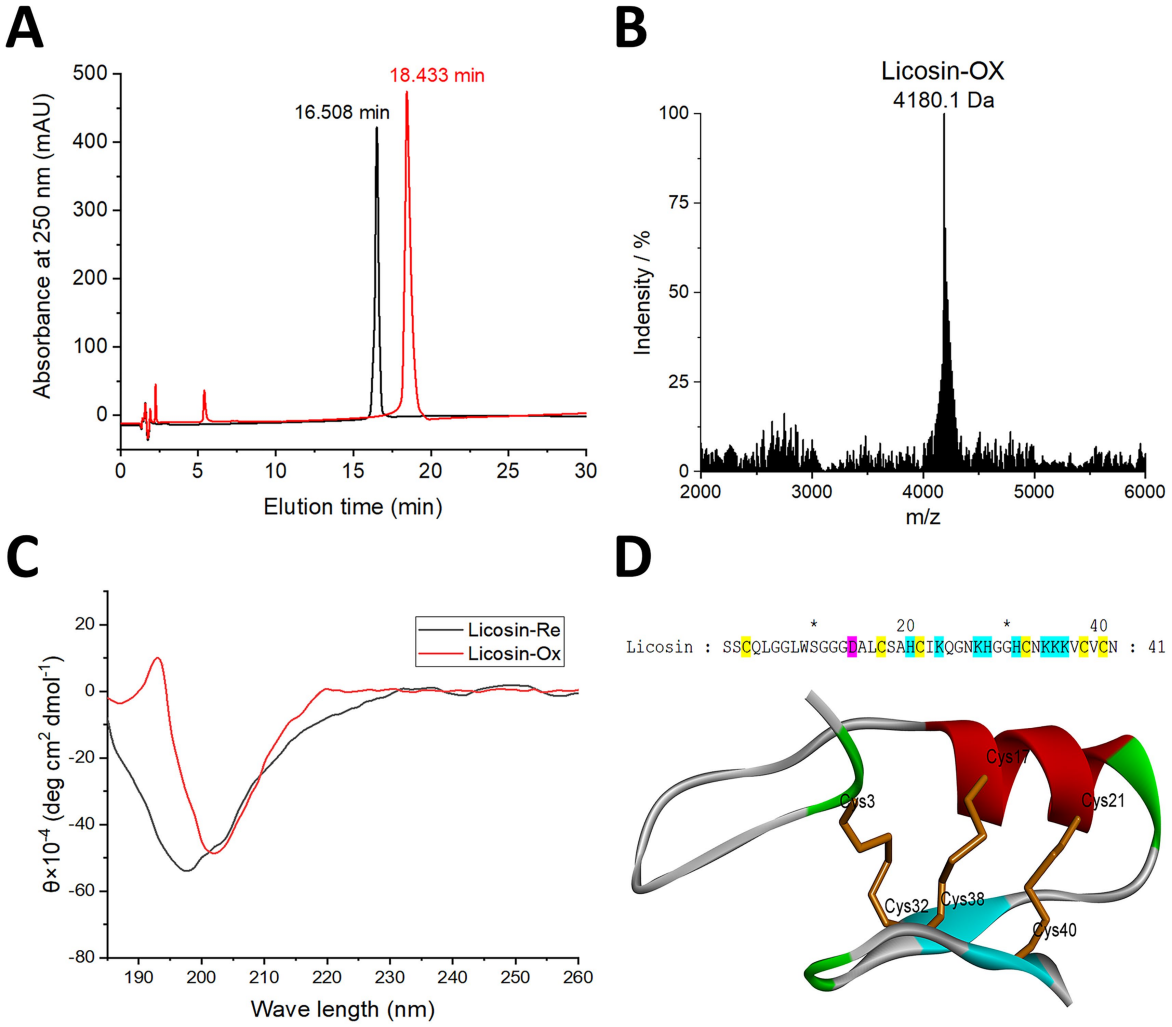
Preparation, mass spectrometry identification, and structure analysis of licosin. **(A)** The analysis of retention time before and after oxidative refolding by RP-HPLC. The absorption of oxidized (Ox) and reduced (Re) peptides at 250 nm is presented as red and black curves, respectively. **(B)** MALDI-TOF MS of the oxidized licosin. **(C)** Secondary structure determination of licosin by CD spectrum. **(D)** The homologous model of licosin. The 3D structures are shown as a solid ribbon model. *α*-Helix, *β*-sheet, and loop areas are colored in red, blue, and green, respectively. The disulfide bonds are shown as a line ribbon, and the cysteine residues are also labeled. Diagrams were generated using Discovery Studio Visualizer Client Version 4.5.

Based on mature licosin, the reduced form of the linear defensin sequence was obtained by solid-phase peptide synthesis. Under slightly alkaline nonionic buffer conditions, disulfide bridges were formed via air oxidation. The cyclized licosin was successfully isolated and purified by RP-HPLC. Before and after disulfide bond pairing and peptide folding, the chromatographic retention times for licosin were 16.508 min and 18.433 min, respectively (Figure 4A). Compared to linear licosin, the spatial conformation of the cyclized peptide, stabilized by intermolecular covalent disulfide bonds, showed lower molecular polarity. Therefore, the retention time of cyclized licosin was longer than that of the linear form. The SDS-PAGE results indicated that there was a single band of licosin peptide, which was located below the 5 KDa protein marker (Supplementary Figure S1). The precise molecular weight of the cyclized licosin, determined by MALDI-TOF MS under reflection mode, was 4180.1 Da (Figure 4B). This was 6.7 Da less than the theoretical molecular weight of the corresponding linear peptide (4186.8 Da). The reduced molecular weight of licosin indicated that it had likely formed three pairs of intramolecular disulfide bridges.

### 3.5 Structural analysis of fungi-derived defensin licosin

The secondary structures of both linear and cyclized licosin were determined by CD spectroscopy. The cyclized defensin presented a conserved cysteine-stabilized *α*-helix and *β*-sheet (CSα/β) scaffold analogous to other fungal defensins and toxins from venomous animals (Wu et al., 2014; Ho et al., 2023), while the linear peptide exhibited a random-coil conformation (Figure 4C). The linear peptide appeared unstructured in aqueous solution with a minimum at ~197 nm. In contrast, a remarkable transition for cyclic licosin was observed, which exhibited a maximum peak at ~193 nm and a minimum at ~202 nm. The red curve indicated a propensity for the presence of α-helical and β-sheet structures. It was suggested that licosin can form a typical CSαβ motif via three intramolecular disulfide linkages. The homology model of licosin was predicted using the Swiss-Model server. The 3D structure was optimized and evaluated by BIOVIA Discovery Studio Visualizer V25^3^. The sequence of the mature peptide of licosin and its spatial structure are shown in Figure 4D. The spatial structure of licosin was mainly composed of one α-helix and two antiparallel β-sheets, and the CSα/β fold was stabilized via the linkage of disulfide bridges (Cys1-Cys4, Cys2-Cys5, and Cys3-Cys6). Based on the phi–psi torsion angles, model quality was validated by the Ramachandran plot. Except for glycine at position 29, 97.56% of the residues of licosin were located in the most favored and allowed regions (Supplementary Figure S2).

### 3.6 *In vitro* antimicrobial effects of licosin

The antimicrobial activities of mature licosin were explored involving both linear and cyclic peptides. Even at a maximum concentration as high as 64 μM, the linear form of licosin displayed no obvious bacteriostatic effects on all tested strains. The MIC values of mature cyclic licosin are shown in Table 1. Licosin exhibited significant antibacterial activities against all reference gram-positive bacteria, with MIC values ranging from 8 to 32 μM. It is noteworthy that it also possessed antibacterial potential against clinical isolates of MRSA and *M. luteus*. Similar to most other cationic fungal defensins (Shen et al., 2020; Li et al., 2024), licosin did not show inhibitory activity against gram-negative bacteria.

**Table 1:** *In vitro* antimicrobial activities of cyclic licosin.

Strains	MIC values/μM	
	Licosin	Ampicillin
Reference gram-positive bacteria		
*Staphylococcus aureus* ATCC6538	16	16
Methicillin-resistant *S. aureus* ATCC 43300	32	> 64
*Staphylococcus epidermidis* AB208187	8	32
*Micrococcus luteus* ATCC 4698	32	32
Reference gram-negative bacteria		
*Escherichia coli* AB94012	> 64	64
*Acinetobacter baumannii* ATCC 19606	> 64	> 64
*Klebsiella pneumoniae* ATCC 31314	> 64	32
Clinical isolates		
Methicillin-resistant *S. aureus*	64	> 64
*Micrococcus luteus*	64	64

### 3.7 Antibacterial mechanism *in vitro*

MRSA ATCC 43300 cells treated with licosin were observed with a SEM to evaluate changes in individual bacteria. As shown in Supplementary Figure S3, the strain in the control group maintained its original form, and the surfaces of the bacteria were smooth and intact. After licosin treatment for 30 min, the morphology of the bacterial cell membrane was significantly disrupted, and the bacteria appeared to shrink and fracture to varying degrees. This further demonstrated that the fungal defensin licosin could achieve bactericidal effects by destroying the cell membrane. Due to the stability of the structure and composition of the bacterial cell membrane, the antibacterial mechanism of membrane disruption can help avoid drug resistance.

The time-kill curve showed that licosin was able to kill all of *S. aureus* ATCC6538 within 4 h (Supplementary Figure S4), demonstrating that licosin possessed strong bactericidal activity. Compared to the positive control of ampicillin, licosin exhibited more potent activity and higher efficiency against *S. aureus* ATCC6538. The primary reason may be that licosin directly disrupts the bacterial membrane, while ampicillin blocks the formation of the cell wall in proliferating strains.

### 3.8 Electrophysiological activities and binding model

**Figure 5:**
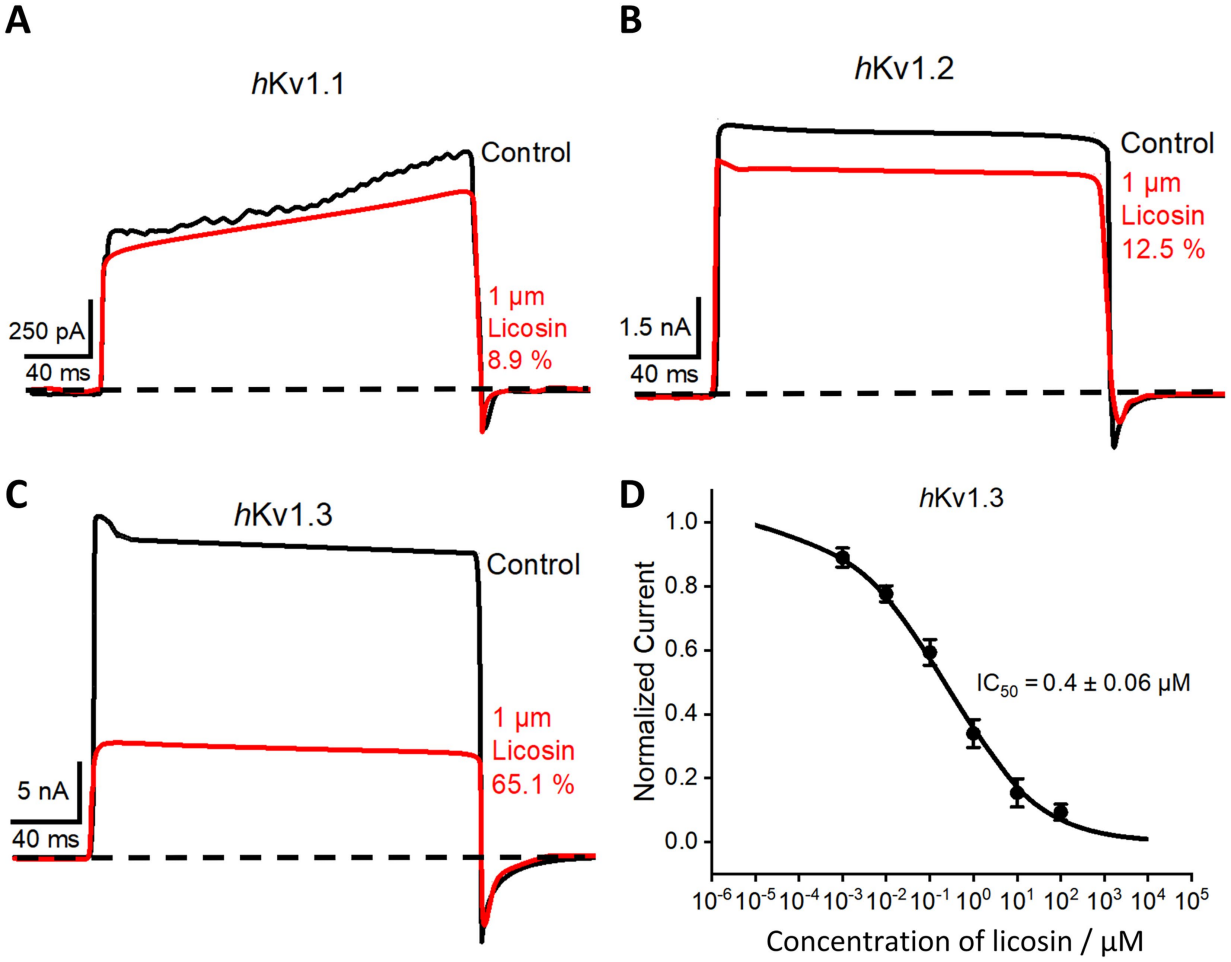
Inhibitory effects of licosin on multiple potassium channels. **(A–C)** Blocking effects of 1 μM licosin on *h*Kv1.1, *h*Kv1.2, and *h*Kv1.3. **(D)** Average normalized current inhibition by various concentrations of licosin for the *h*Kv1.3 channel. Hill equation fitting gives an IC_50_ value of 0.40 ± 0.06 μM.

The identified sequences and homologous 3D structure of licosin indicated that it could be a toxin-like molecule acting on ion channels. To verify our hypothesis, the electrophysiological technique of whole-cell patch-clamp recording was adopted to explore the effects of licosin on various human potassium ion channels, including *h*Kv1.1, *h*Kv1.2, and *h*Kv1.3. The subunits of these channel proteins were overexpressed and successfully assembled into functional K + channels in transfected HEK293 cells. Licosin showed weak effects on *h*Kv1.1 and *h*Kv1.2 channels, with inhibitory ratios of 8.9 and 12.5%, respectively (Figures 5A,B). Encouragingly, it demonstrated a pronounced inhibitory effect on the *h*Kv1.3 channel with a 65.1% inhibitory ratio at a concentration of 1 μM (Figure 5C). A series of concentrations of licosin were prepared to explore the dose-dependent response against the *h*Kv1.3 channel. The dose–effect curve was fitted to the Hill equation, yielding an IC50 value of 0.4 ± 0.06 μM (Figure 5D). These results proved that licosin had significant blocking potency and good specificity for the *h*Kv1.3 channel. The linear peptide displayed no inhibitory effects on all tested potassium channels, indicating that the spatial structure based on CS/αβ architecture played a key role in exerting its biological functions.

**Figure 6:**
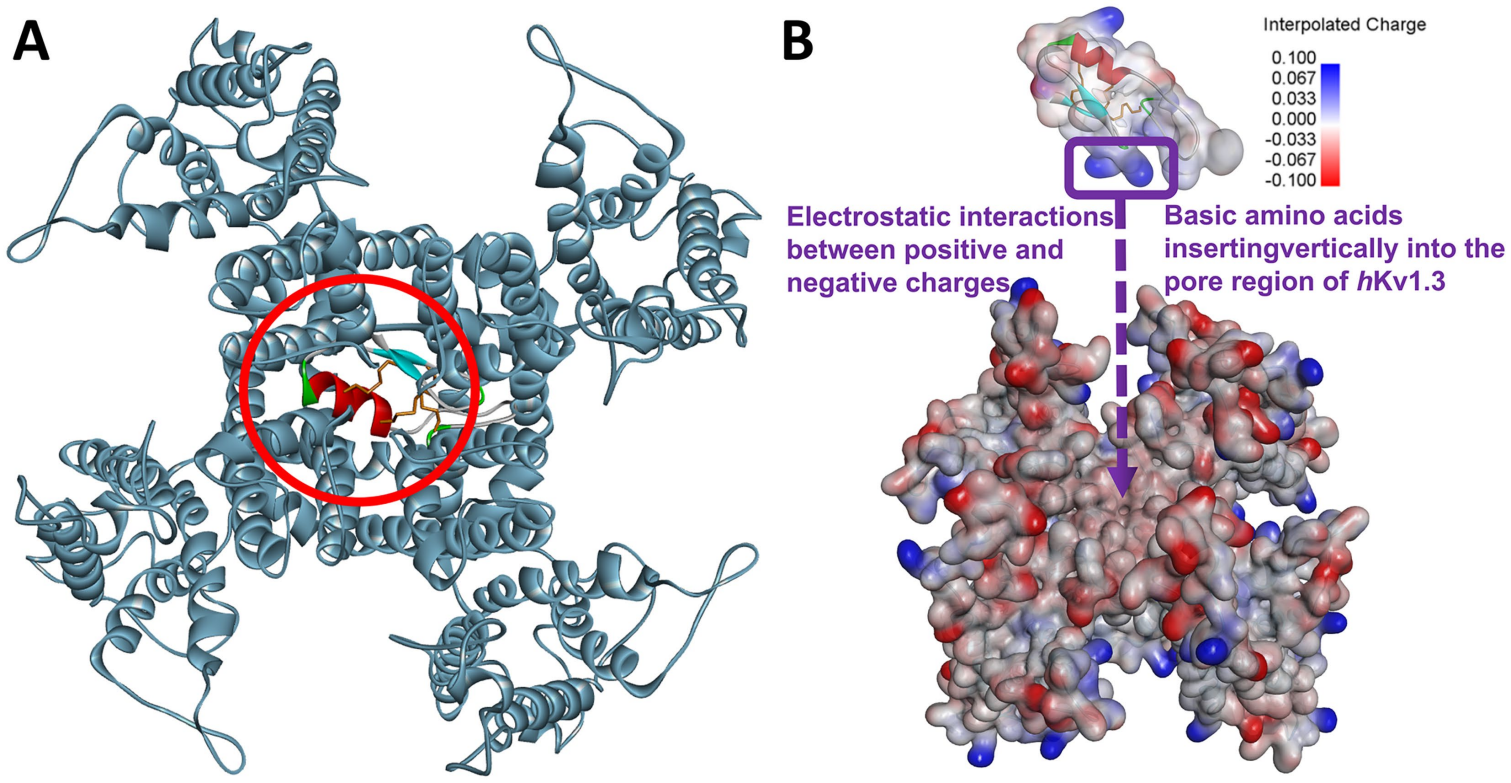
Binding model between licosin and *h*Kv1.3 potassium channel. **(A)** Overall top view of molecular interactions between licosin and *h*Kv1.3. The voltage-gated potassium channel *h*Kv1.3 is shown as a solid ribbon model and colored dark gray green. The pore region of the target (*h*Kv1.3) and the ligand (licosin) is indicated by a purple circle. **(B)** The pattern of electrostatic interaction between licosin and the pore region of *h*Kv1.3. The surfaces of basic and acidic residues on licosin are colored blue and red, respectively. The surfaces with negative and positive partial charges on the pore region of hKv1.3 are also colored blue and red, respectively.

The ZDOCK program, which can search for all possible interaction sites between the channel protein and the defensin peptide via translation and rotation, was used to predict the binding model using an energy-based scoring function (Vreven et al., 2011; Pierce et al., 2014). To reduce the calculation time, the ligand licosin and the target *h*Kv1.3 channel were treated as flexible and rigid bodies, respectively. The fungal-derived licosin peptide preferentially binds in the pore region, with the *ε*-amino group of the side chain of lysine 34 inserting into the open pore to further obstruct the passage of potassium ions (Figure 6A). Through spatial electrostatic interactions between licosin and the *h*Kv1.3 protein, the basic amino acids of licosin at the C terminus, which carry positive partial charges, plugged vertically into the negatively charged pore region of the channel protein (Figure 6B). The docking model and interaction mode revealed that the key basic groups of licosin (two lysine residues at positions 34 and 35) are critical amino acids contributing to the blockage of the *h*Kv1.3 channel by three acidic aspartate residues at positions 422, 423, and 433.

### 3.9 The structure–activity relationship of liconsin peptide

To further explore the acting site of licosin and its structure–activity relationship, alanine scanning of the key amino acids was carried out, and five mutants were designed (Supplementary Figure S5). Compared to the wild type licosin, the antibacterial activities against *S. aureus* ATCC6538 and *h*Kv1*.3* inhibition effects of the five mutants are presented in Supplementary Table S2. The results indicated that two basic lysine residues at positions 23 and 27 contributed to the antimicrobial effects. Since the cell wall of *S. aureus* is negatively charged, these two basic lysines can interact with the positively charged licosin (Supplementary Figure S6). The inhibitory rates of these five mutants revealed that three lysine residues at positions 34 to 36 were related to the blocking effects on *h*Kv1*.3* ion channels. Consistent with the results of molecular docking (Figure 6), the two lysines at positions 34 and 35 were the key amino acids interacting with the target of *h*Kv1*.3*. Therefore, the basic lysine residues in different regions exert antimicrobial (Lys 23 and 27) and *h*Kv1*.3* channel inhibitory (Lys 34, 35, and 36) effects, respectively.

### 3.10 Cell viability *in vitro*

The cytotoxic effects of licosin on HUVEC and NIH-3 T3 cells were determined by the CCK-8 assay. As shown in Supplementary Figures S7, S8, the licosin peptide exhibited no cytotoxicity toward these two cell lines. Interestingly, licosin can promote the proliferation of HUVEC cells at concentrations up to 64 μM. These findings hold significant implications for potential therapeutic applications in the future, as they suggest that the licosin peptide is biocompatible and capable of supporting endothelial cell growth at high concentrations. The absence of toxicity in NIH-3 T3 cells further enhances its safety profile, making it a promising candidate for various biomedical applications. Additionally, the safety of licosin for HUVEC cells lays a solid foundation for injection applications.

### 3.11 The effect of pH and salt on the stability

The results of salt and pH stability of licosin are displayed in Supplementary Table S3. Licosin retained stable antibacterial activity against *S. aureus* ATCC6538 in the NaCl range of 0–200 mM. Meanwhile, it exhibited stable activity against the tested strain over a wide range of pH values (pH 5.0–9.0). However, in stronger acidic (pH 4.0) and alkaline (pH 10.0) environments, licosin showed no antibacterial effects at the maximum test concentration of 64 μM.

## 4 Discussion

Recently, nosocomial infections caused by invasive fungi have shown an upward trend and become a severe problem. Patients with cancer after treatment are always a susceptible population. In this research, a clinical pathogenic fungus *L. corymbifera* was isolated and identified. Unlike most fungal defensin genes that contain introns (Mygind et al., 2005; Zhu et al., 2012; Shen et al., 2020), the licosin gene had no introns, consisting of 207 nucleic acid bases coding for 68 amino acids and a terminator codon. Most defensin peptides are initially synthesized as prepro-peptides, typically consisting of three distinct segments. The signal peptide is an N-terminal sequence (about 20 amino acids) that directs the peptide to the endoplasmic reticulum, where it is rapidly cleaved during translation. The pro-peptide can prevent premature membrane disruption or autotoxicity and also aids in proper folding. The mature peptide is the final, biologically active domain containing 18 to 45 amino acids stabilized by conserved intramolecular disulfide bonds. Through sequence alignment with other known defensins (Figure 2C) and mass spectrometry analysis (Figure 3C), the cleavage site for the pro- and mature peptide was identified. For the licosin peptide, the demarcation points of the signal peptide and pro−/mature peptide were A20/S21 and R27/S28, respectively. It also presented similar characteristics to other defensins and toxins, involving six or eight highly conserved cysteine residues (Craik et al., 2001; Zhu, 2008).

Due to the low level of defensin expression in fungi, it is very difficult to isolate the licosin peptide from the total protein extracted from *L. corymbifera*. Based on the amino acid sequence confirmed by LC-ESI-Q-TOF-MS/MS, the linear peptides of licosin were obtained through solid-phase synthesis. Through air exposure and oxidation in Tris–HCl buffer, the chemical entity of defensin with three pairs of intramolecular disulfide linkages was successfully obtained. In the process of each S–S bond formation, two hydrogen atoms were removed. Hence, the number of intramolecular disulfide bridges can be easily calculated. For the licosin peptide containing six cysteines, it was indicated that all sulfhydryl groups were involved in the formation of the disulfide bonds.

The research on the biological effects of licosin defensin has revealed that it is a bifunctional molecule with antimicrobial and potassium channel-blocking activities. Unlike typical defensins with antibacterial effects (Ma et al., 2020), licosin simultaneously exhibited both antimicrobial activities and a selective inhibitory effect on the *h*Kv1.3 potassium channel, similar to animal toxins (Qin et al., 2021). The sequence alignment between the licosin peptide and Kv1.3-related animal toxins derived from venomous animals indicated a similarity of 25.1 to 35.4%. The disulfide bonding pattern of licosin was the same as that of three toxins from sea anemones, scorpions, and spiders, but different from the jellyfish toxin (Supplementary Figure S9). Licosin also displayed great potential against gram-positive bacteria, including clinical isolates of MRSA.

For the clinical fungi *L. corymbifera*, obtaining sufficient nutrients and resisting the host’s immune system are the two essential conditions for successfully infecting human hosts. Two strains, *S. aureus* and *M. luteus*, are common colonizing bacteria on the human skin surface (Piewngam and Otto, 2024). Once the human Kv1.3 potassium channel, recognized as a target for immune regulation, is blocked, the immune response can be inhibited (Matkó, 2023). Hence, the toxin-like defensin licosin might play an essential and irreplaceable role during the processes of infection and colonization.

The 3D model of licosin was successfully constructed using homology modeling methods. Regarding antimicrobial or *h*Kv1.3 channel inhibitory activities, only the oxidized form with three pairs of intramolecular disulfide bonds can exert corresponding biological functions. Structurally, licosin with three disulfide linkages primarily consists of one *α*-helix, two antiparallel *β*-sheets, and two loop regions (Figure 4D). To date, most fungal defensins are mainly stabilized by three pairs (such as plactasin, micasin, and triiinsin), four pairs (such as purlisin-CT), or six pairs (such as copsin) of disulfide bonds (Mygind et al., 2005; Zhu et al., 2012; Essig et al., 2014; Shen et al., 2018, 2020), indicating that the CSαβ motif determines the spatial conformation of these defensin peptides. The exploration of the interaction mode between licosin and the *h*Kv1*.3* target through Z-dock software suggested that the electrostatic attractive force might induce and enhance the affinity between the *h*Kv1*.3* binding domain, which has negative charges, and the basic residues near the C terminus of licosin, which have high densities of positive charges. It exhibited higher affinity and remarkable blocking activity against the *h*Kv1*.3* channel.

## 5 Conclusion

In summary, a new fungal defensin termed licosin was discovered from the human pathogenic fungi *L. corymbifera*, which was identified at both the nucleic acid and protein levels. After the removal of the signal peptide, the precursor peptide of defensin was processed into the mature peptide, namely licosin. It exhibited antibacterial activities against gram-positive bacteria and potassium channel blocking ability. The dual-functional licosin could be further explored as a potential therapeutic agent for bio-drug research and development.

## Data Availability

The original contributions presented in the study are included in the article/Supplementary material, further inquiries can be directed to the corresponding author.

## Footnotes

1
http://www.cbs.dtu.dk/services/SignaIP

2
http://swissmodel.expasy.org

3
https://discover.3ds.com/discovery-studio-visualizer-download

## Supplementary material

SUPPLEMENTARY FIGURE S1 SDS-PAGE gels of licosin peptide.

SUPPLEMENTARY FIGURE S2 Ramachandran map of modeled licosin peptide. The circles represent the amino acid residues other than glycine and proline, and the triangles represent the glycine residues.

SUPPLEMENTARY FIGURE S3 Scanning electron microscopic images of MRSA ATCC 43300 treated with licosin. **(A)** Negative control. Bacterial cells had regular and spherical morphology, with plump and smooth surface, which were uniform in size and distribution. **(B)** 30 min after licosin treatment. MRSA cells presented irregular, withered, and coarse surfaces, forming aggregations and adhesions. Furthermore, these cells were not uniform in size and distribution.

SUPPLEMENTARY FIGURE S4 Time-kill curve of fungal defensin licosin against *S. aureus* ATCC6538

SUPPLEMENTARY FIGURE S5 Five mutants designed via alanine scanning of all basic lysine residues in licosin

SUPPLEMENTARY FIGURE S6 The key amino acids related to the antibacterial and ion channel inhibitory activities of licosin

SUPPLEMENTARY FIGURE S7 Cell viability of HUVEC cells exposed to licosin or ampicillin in different concentrations.

SUPPLEMENTARY FIGURE S8 Cell viability of NIH-3T3 cells exposed to licosin or ampicillin in different concentrations.

SUPPLEMENTARY FIGURE S9 Multiple sequence alignments of licosin. The sequence homology between licosin and animal toxins as well as their disulfide patterns. The cysteines, acidic residues, and basic residues were colored in brilliant yellow, magenta and blue, respectively.
