# Supplementary figures and images for: Licosin, a multifunctional defensin peptide originated from the clinical fungus Lichtheimia corymbifera with antibacterial and potassium ion channel blocking effects
Source: Front Microbiol. 2026 May 11;17:1808106. doi: 10.3389/fmicb.2026.1808106 (PMC13199095; doi:10.3389/fmicb.2026.1808106)

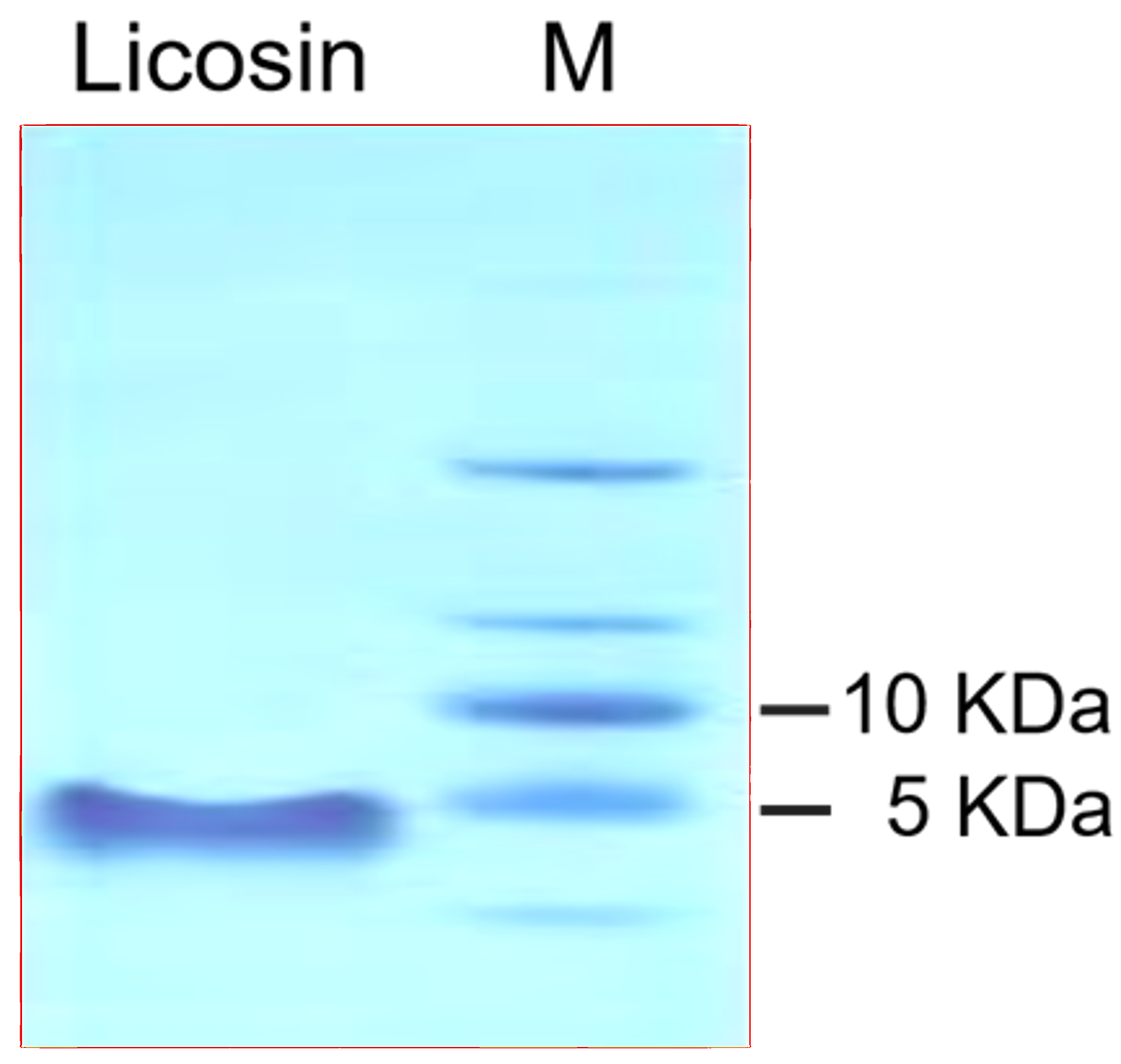

Supplement: SUPPLEMENTARY FIGURE S1 — SDS-PAGE gels of licosin peptide. [file Image_1.TIF]

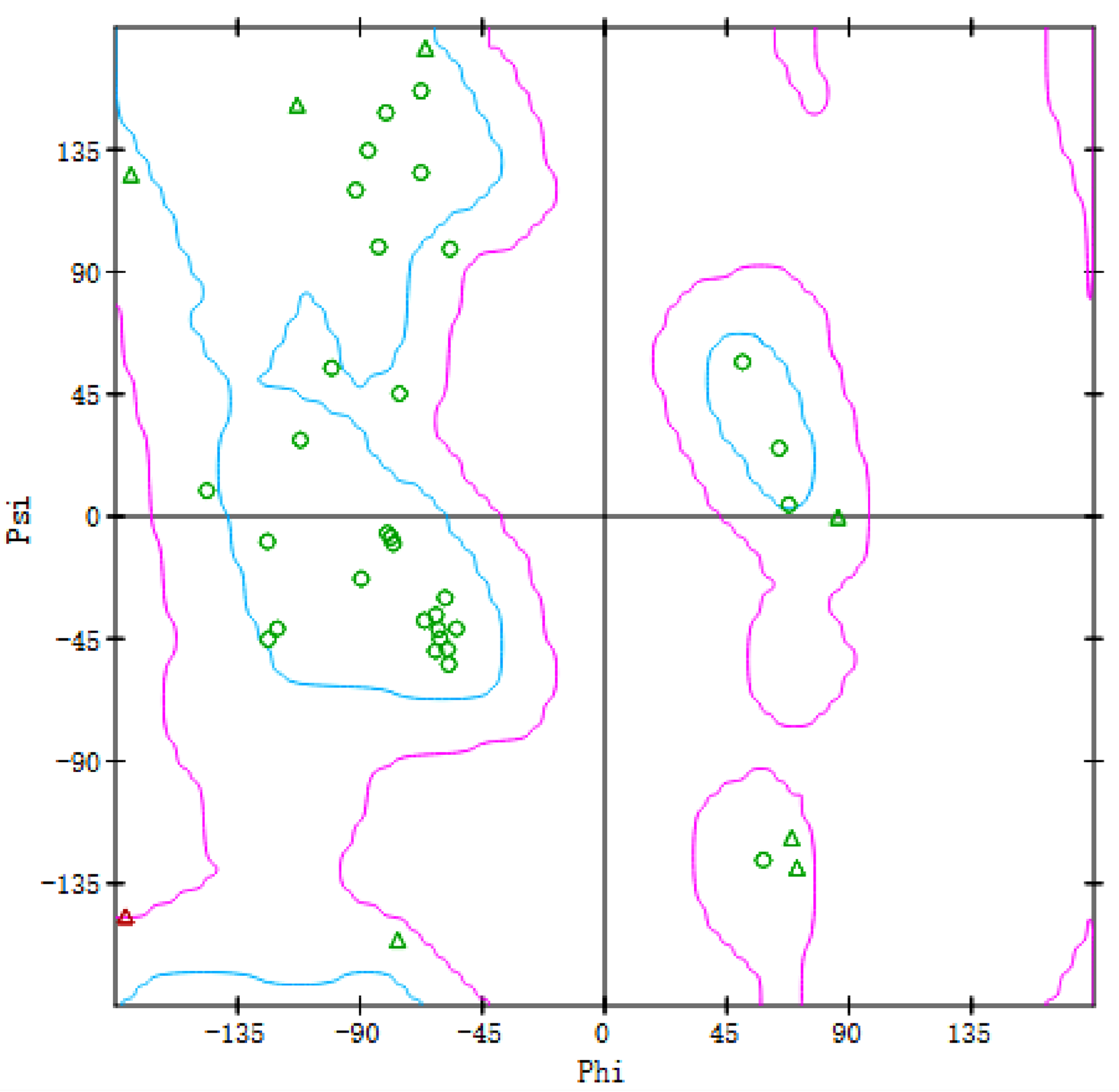

Supplement: SUPPLEMENTARY FIGURE S2 — Ramachandran map of modeled licosin peptide. The circles represent the amino acid residues other than glycine and proline, and the triangles represent the glycine residues. [file Image_2.TIF]

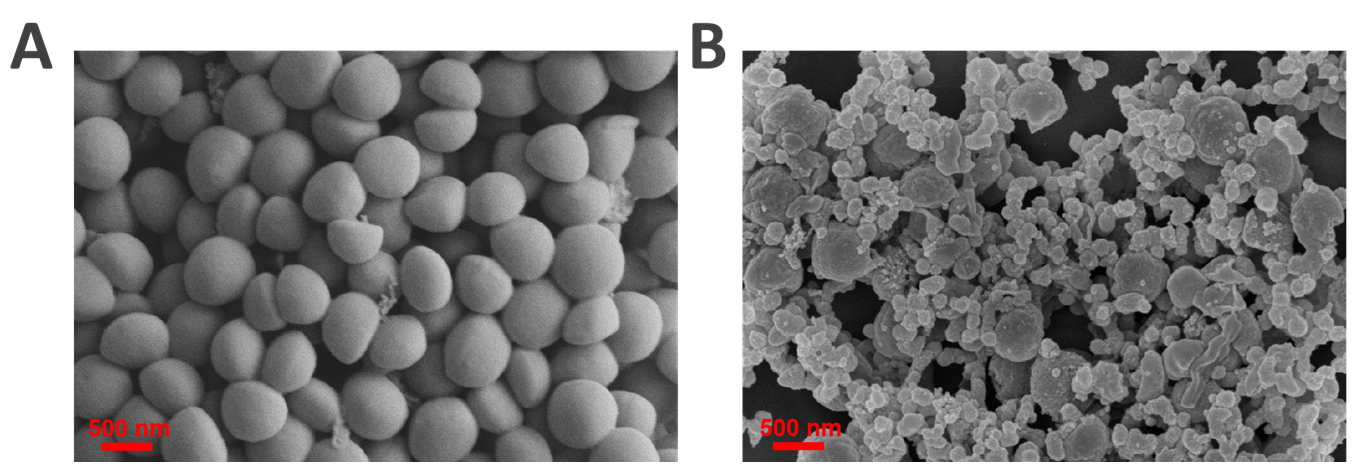

Supplement: SUPPLEMENTARY FIGURE S3 — Scanning electron microscopic images of MRSA ATCC 43300 treated with licosin. (A) Negative control. Bacterial cells had regular and spherical morphology, with plump and smooth surface, which were uniform in size and distribution. (B) 30 min after licosin treatment. MRSA cells presented irregular, withered, and coarse surfaces, forming aggregations and adhesions. Furthermore, these cells were not uniform in size and distribution. [file Image_3.TIF]

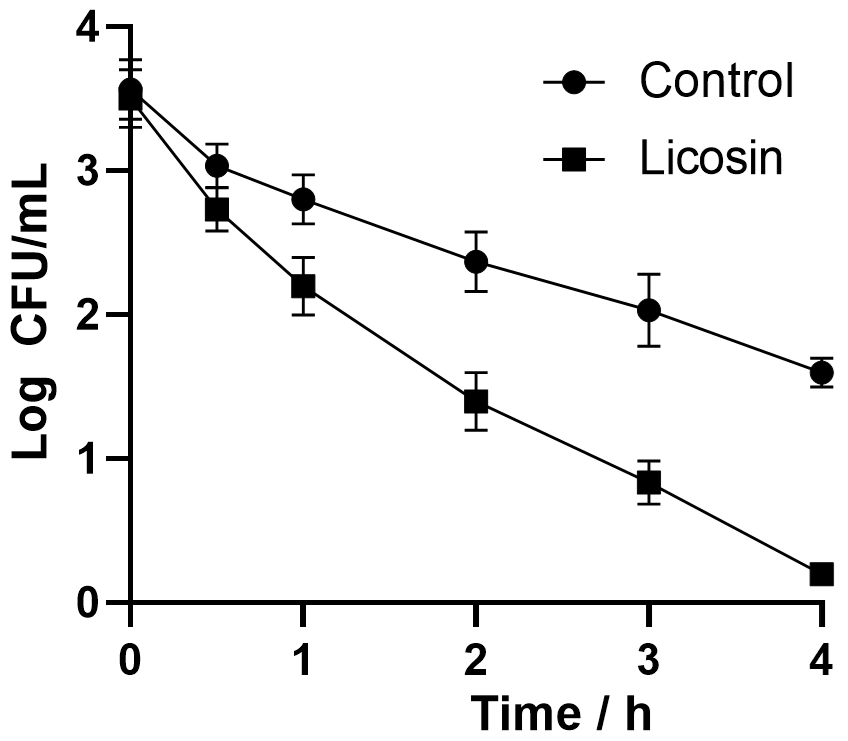

Supplement: SUPPLEMENTARY FIGURE S4 — Time-kill curve of fungal defensin licosin against S. aureus ATCC6538 [file Image_4.TIF]

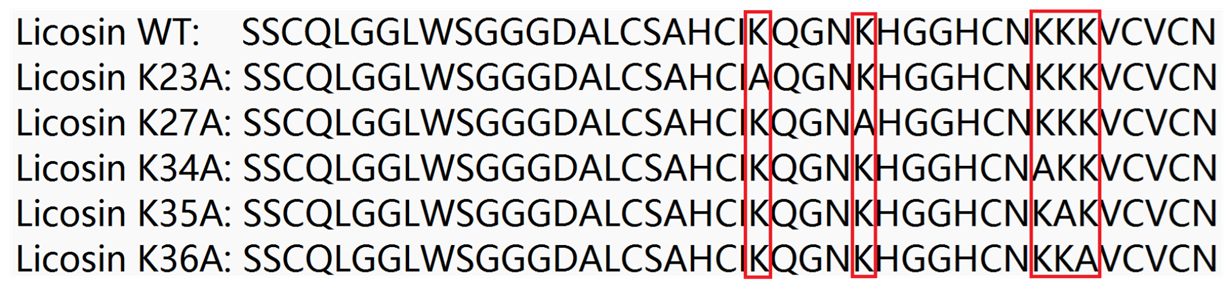

Supplement: SUPPLEMENTARY FIGURE S5 — Five mutants designed via alanine scanning of all basic lysine residues in licosin [file Image_5.TIF]

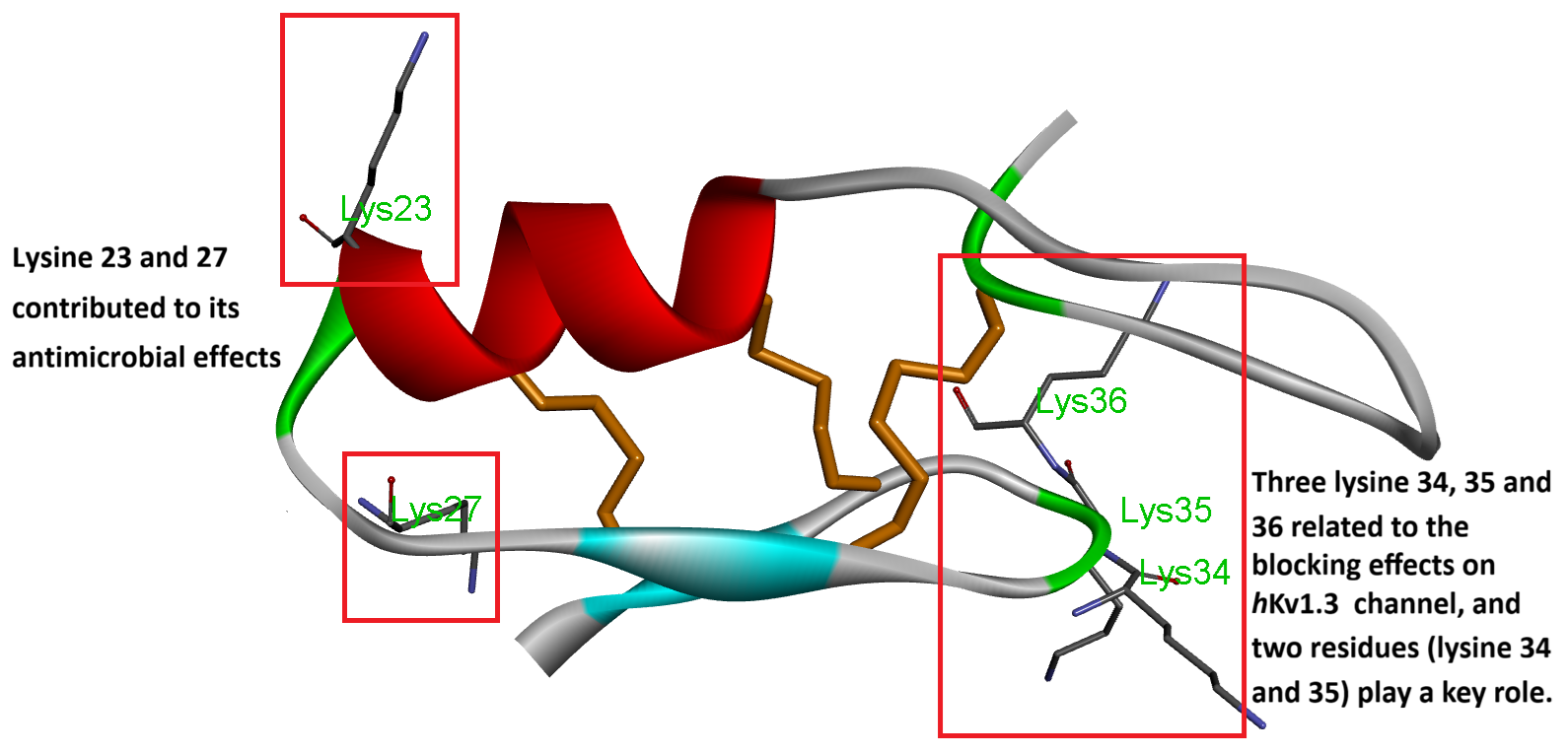

Supplement: SUPPLEMENTARY FIGURE S6 — The key amino acids related to the antibacterial and ion channel inhibitory activities of licosin [file Image_6.TIF]

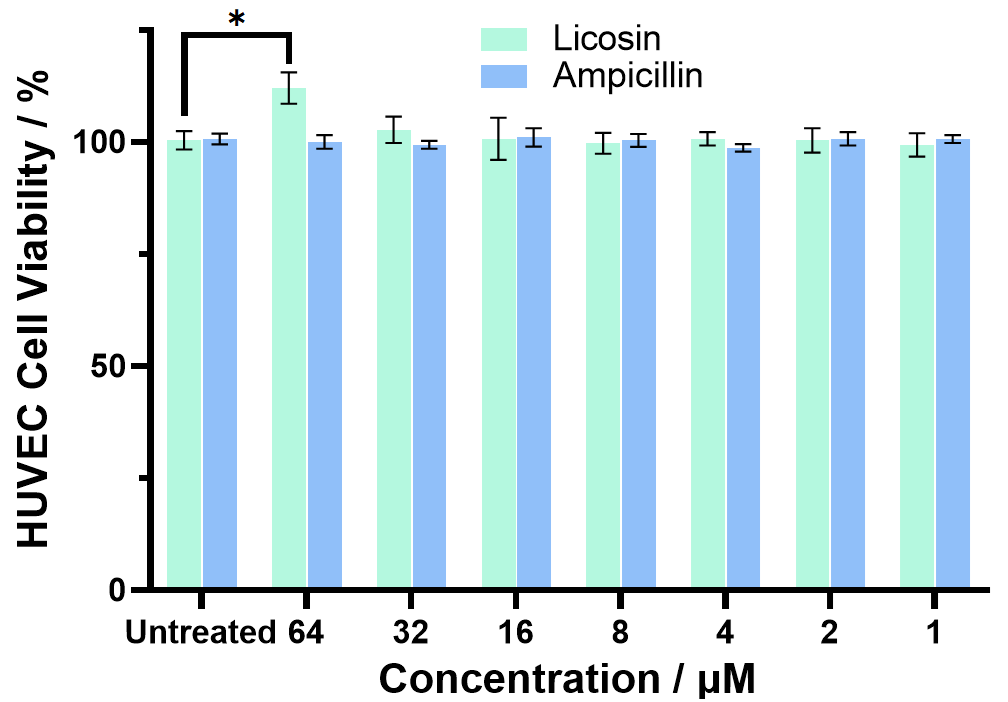

Supplement: SUPPLEMENTARY FIGURE S7 — Cell viability of HUVEC cells exposed to licosin or ampicillin in different concentrations. [file Image_7.TIF]

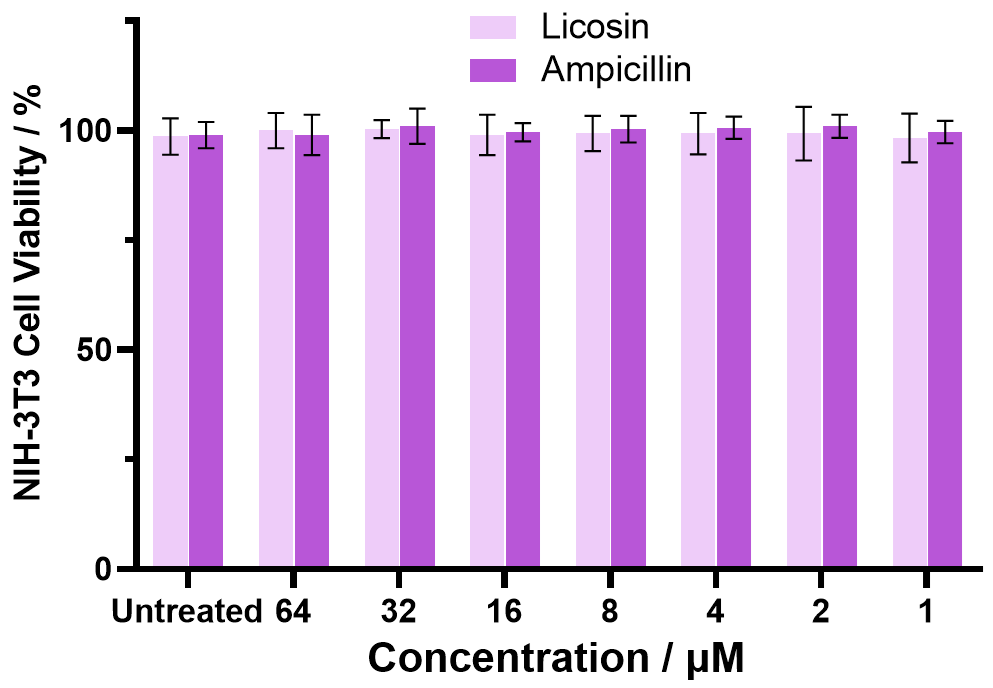

Supplement: SUPPLEMENTARY FIGURE S8 — Cell viability of NIH-3T3 cells exposed to licosin or ampicillin in different concentrations. [file Image_8.TIF]

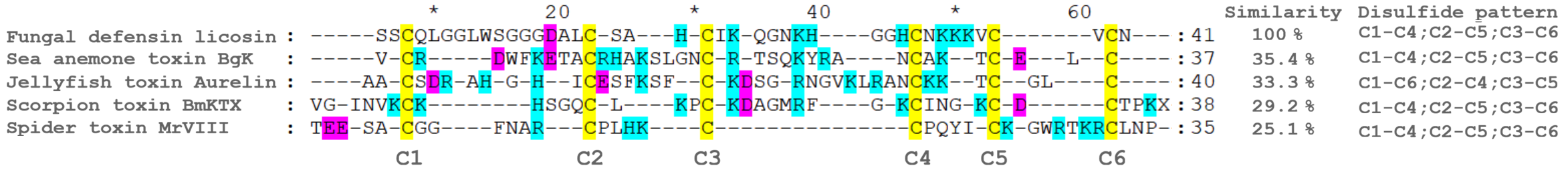

Supplement: SUPPLEMENTARY FIGURE S9 — Multiple sequence alignments of licosin. The sequence homology between licosin and animal toxins as well as their disulfide patterns. The cysteines, acidic residues, and basic residues were colored in brilliant yellow, magenta and blue, respectively. [file Image_9.TIF]
