# Supplementary material for: Licosin, a multifunctional defensin peptide originated from the clinical fungus Lichtheimia corymbifera with antibacterial and potassium ion channel blocking effects
Source: Front Microbiol. 2026 May 11;17:1808106. doi: 10.3389/fmicb.2026.1808106 (PMC13199095; doi:10.3389/fmicb.2026.1808106)
Supplement: Supplementary file 10 [file Table_1.DOCX]

**Supplementary Table S1** The coverage rates of mapping transcriptome sequence into genome data

| **Strains** | **GenBank ID** | **Coverage rates / %** |
| --- | --- | --- |
| *L. corymbifera* B2541 | GCA_000697475.1 | 97.24 |
| *L. corymbifera* | GCA_023629935.1 | 96.45 |
| *L. corymbifera* | GCA_037042095.1 | 98.01 |
| *L. corymbifera* JMRC:FSU:9682 | GCA_000723665.1 | 96.23 |
| *L. corymbifera* 008-049 | GCA_000697175.1 | 98.38 |
