# Supplementary material for: Licosin, a multifunctional defensin peptide originated from the clinical fungus Lichtheimia corymbifera with antibacterial and potassium ion channel blocking effects
Source: Front Microbiol. 2026 May 11;17:1808106. doi: 10.3389/fmicb.2026.1808106 (PMC13199095; doi:10.3389/fmicb.2026.1808106)
Supplement: Supplementary file 11 [file Table_2.DOCX]

**Supplementary Table S2** The antibacterial and *h*Kv1.3 channel inhibitory activities of the five mutants of licosin peptide

| **Peptide** | **MIC values / μM** | ***h*Kv1.3 inhibitory rate / %** |
| --- | --- | --- |
| Licosin WT | 16 | 65.1 |
| Licosin K23A | 64 | 60.4 |
| Licosin K27A | > 64 | 63.6 |
| Licosin K34A | 16 | 17.2 |
| Licosin K35A | 32 | 20.0 |
| Licosin K36A | 16 | 49.8 |
