# Supplementary material for: Licosin, a multifunctional defensin peptide originated from the clinical fungus Lichtheimia corymbifera with antibacterial and potassium ion channel blocking effects
Source: Front Microbiol. 2026 May 11;17:1808106. doi: 10.3389/fmicb.2026.1808106 (PMC13199095; doi:10.3389/fmicb.2026.1808106)
Supplement: Supplementary file 12 [file Table_3.DOCX]

**Supplementary Table S3** Effects of salt and pH on activity of licosin

| ***S. aureus* ATCC6538** | **MIC values / μM** |
| --- | --- |
| **NaCl concentration / μM** | |
| 0 | 16 |
| 50 | 16 |
| 100 | 16 |
| 150 | 32 |
| 200 | 64 |
| 250 | > 64 |
| **pH value** |  |
| 4 | > 64 |
| 5 | 64 |
| 6 | 16 |
| 7 | 16 |
| 8 | 16 |
| 9 | 32 |
| 10 | > 64 |
